# Stem-Cell Derived Exosomal microRNAs as Biomarkers and Therapeutics for Pediatric Cardiovascular Disease

**DOI:** 10.1007/s11936-025-01088-0

**Published:** 2025-04-09

**Authors:** Aaron H. Wasserman, Bana Abolibdeh, Reema Hamdan, Charles C. Hong

**Affiliations:** 1 Department of Medicine, Michigan State University College of Human Medicine, East Lansing, MI USA; 2 Henry Ford Health + Michigan State Health Sciences, Detroit, MI USA

**Keywords:** Cardiovascular Disease, Congenital Heart Disease, Mesenchymal Stem Cells, MicroRNAs, Non-Coding RNAs, Stem Cell-Derived Exosomes

## Abstract

**Purpose of Review:**

In recent years, several pre-clinical studies have demonstrated the therapeutic potential of stem cell-derived exosomes in the treatment of cardiovascular disease (CVD). Here, we evaluate their potential as biomarkers for the detection and monitoring of CVD, with a particular focus on pediatric heart disease.

**Recent Findings:**

Exosomes isolated from stem cell sources, including mesenchymal stem cells (MSCs) and pluripotent stem cells (PSCs), benefit cardiovascular function, inflammatory responses, and angiogenesis in injured and diseased hearts. These exosomes carry a variety of cargo, such as proteins, lipids, and nucleic acids. However, the majority contain non-coding RNA molecules.

**Summary:**

Review of the existing literature for several non-coding RNAs and their relationship to CVD suggests that exosomes containing microRNAs (miRNAs) can serve as promising biomarkers for CVD due to their presence in circulation, ease of isolation, and therapeutic potential. These biomarkers are especially promising as screening and diagnostic tools for the early detection of pediatric and congenital heart disease.

## Opinion Statement

In recent years, the pre-clinical literature on the potential therapeutic benefits of stem cell-derived exosomes has grown exponentially. Naturally circulating exosomes are relatively easy to isolate from circulation and make for promising engineered constructs that can carry a variety of cargo, including proteins, lipids, nucleic acids, metabolites, and other bioactive molecules. In addition, their safety, stability, and low immunogenicity make them promising structures for targeted therapeutic delivery nearly anywhere in the body, hence why several clinical trials are underway in this realm.

Here, we discuss the potential of stem cell-derived exosomes as more than just therapeutics for cardiovascular disease, but also potential biomarkers. Specifically, we believe they show promise in the early detection of pediatric heart disease. Their documented beneficial effects in several cardiac pathologies combined with their presence during gestation suggest they could be used to monitor for congenital heart disorders. In addition, the correlation between maternal placental exosomal cargo and congenital cardiac defects demonstrates the importance of using exosomes as a biomarker for these disorders.

Theoretically, any exosomal cargo molecule could serve as an effective biomarker. We believe microRNA molecules present the most promising candidates due to their specific associations with individual heart disorders and their high stability and easy accessibility. In addition, several methods already exist for their detection in cells, tissues, and blood samples. Overall, we suggest stem cell-derived exosomal microRNAs be utilized in combination with existing detection methods, such as pulse oximetry and echocardiography, for the earliest and most effective screening for pediatric heart disease.

## Introduction

Extracellular vesicles (EVs) are nano- to micro- sized lipid bilayer membrane encapsulated particles released by all cell types. Once secreted, they fuse or interact with recipient cells, inducing cell–cell interactions or long-range signaling pathways [1–4]. Recently, the International Society of Extracellular Vesicles (ISEV) recommended specific terms for different types of identified EVs. In the literature, small EVs (sEVs) are mostly referred to as exosomes and large EVs (lEVs) are described as microvesicles (MVs), microparticles (MPs), ectosomes, oncosomes, and apoptotic bodies [3, 5]. EVs contain proteins, lipids, metabolites, and nucleic acids such as DNA, microRNA, and messenger RNA (mRNA) [4, 5]. They are characterized by their cargo content, their size, as well as their origin. Small EVs range from 50–200 nm and originate from endosomes; Large EVs range from 200–1,000 nm and are derived from the plasma membrane; Apoptotic bodies range from 500–10,000 nm and are released as blebbing cells undergo apoptosis [2, 5]. On the smallest scale, exomeres, a newly discovered type of EV, are sized < 50 nm and are involved in metabolic regulation, signaling, and immune modulation [6]. Naturally released EVs modulate several processes in the body through their cargo composition, influencing cell signaling pathways. Such processes include homeostasis maintenance, pro- and anti-inflammatory functions, immune responses, angiogenesis, tissue repair, neuron communication, and cardiovascular physiology [7–9]. EVs have been highlighted for their immense potential to serve as biomarkers and therapeutic agents for various disorders such as cancer, neurodegenerative disease, diabetes, viral infections, autoimmune disease, and cardiovascular disease (CVD) [3, 10, 11].

One class of small EV that has garnered much attention recently is exosomes (~ 40–160 nm). The biogenesis of exosomes is a tightly regulated process starting with integration of early-sorting endosomes into late-sorting endosomes, forming multivesicular bodies (MVBs). These structures contain intraluminal vesicles (ILVs) that eventually become exosomes. Exosomes are enriched for specific biomarkers, thereby distinguishing them from other MVs [5]. These sEVs carry signals generated intracellularly to the extracellular space of specific cells throughout the body. Furthermore, under pathological conditions, cells release a customized set of exosomes with functional characteristics reflecting the disease phenotype [1, 5]. Most cell types in the body can produce exosomes. In the heart, this includes cardiomyocytes, endothelial cells, fibroblasts, platelets, smooth muscle cells, monocytes, and macrophages [5]. Each cell type releases exosomes carrying a unique set of cargo, including proteins, lipids, and nucleic acids, to inform of cellular conditions and determine downstream targets [12]. In this review, we will discuss the roles of exosomes in CVD and critically evaluate the existing literature on several exosome cargo types. We will also consider their potential as biomarkers for CVD, with a focus on pediatric congenital heart disease (CHD).

### Extracellular Vesicles in Cardiovascular Disease

CVD is the top cause of global and national mortality placing a strain on healthcare systems leading to substantial economic and societal burdens. In the United States alone, ~ 128 million Americans over the age of 20 have some type of CVD including coronary heart disease, heart failure, stroke, or hypertension, accounting for ~ 48.6% of the population [13]. CVD is not limited to adults, as CHD affects ~ 1% of all live births and is one of the leading causes of infant mortality, attributed to > 200,000 deaths annually [13, 14]. It is estimated that 13.3 million people globally are living with a congenital cardiac defect, with long-term complications including arrhythmias, endocarditis, and heart failure, as well as neurodevelopmental defects and mental health disorders, all resulting in high mortality rates and lower quality of life [13, 15, 16]. While CHD is presently classified according to gross anatomical abnormalities, it is increasingly recognized that these disorders are caused by diverse distinct molecular and cellular defects, and physiological derangement [17]. Therefore, development of mechanism-based diagnosis methods individualized for each CHD patient is critical.

Circulating cardiac EVs, specifically exosomes, are found in bodily fluids such as blood, urine, saliva, cerebrospinal fluid, synovial fluid, amniotic fluid, and breast milk. They play various roles ranging from supporting normal cardiovascular functions and heart development to serving as a central pathological mechanism in CVD [3, 4]. Increasing evidence suggests EVs play a role in maintaining normal cardiac structure and function under physiological conditions and disease states, accomplishing this by altering their constituent cargo molecules [1, 18, 19]. To further highlight this application and potential, a study derived three subpopulations of cardiac progenitor cell-derived sEVs with unique protein profiles and assayed them for repair related processes. The middle and smaller sized sEVs exhibited pro-angiogenic and anti-fibrotic activity, with the middle population enriching for an exosomal protein profile [20]. This work highlights the diversity of this population of sEVs and its mechanism of action and helps to narrow down which sEVs may have clinical relevance.

Stem-cell derived EVs are a promising avenue for developing novel personalized CVD therapies. These approaches leverage the regenerative properties of stem cells and the natural tendency of EVs to target cardiac cells [21]. Exosomes derived from mesenchymal stem cells (MSCs) can be beneficial in treating CVD due to their biocompatibility, stability, ease of isolation, and presence in circulation. In addition, they serve as a potential cell-free system for safe and targeted delivery of drugs, thereby circumventing the limitations of stem cell-based therapy, such as storage constraints and immunogenicity [22, 23]. In one recent landmark study, exosomes were isolated from stem cells and administered to both mice and pigs after myocardial infarction (MI) via use of a nebulizer. Notably, the exosomes reached the heart within 1 h, with high retention at 48 h after repeated delivery. Mice that inhaled the exosomes showed increased survival, higher left ventricular function, reduced cardiac fibrosis, and increased cardiomyocyte (CM) proliferation. This method, referred to as SCENT (stem cell-derived exosome nebulization therapy), also provided cardioprotection and functional benefits to injured pig hearts, likely through downregulation of CD36 in endothelial cells [24]. Several other studies in the past two years have demonstrated the benefits of stem cell-derived exosomes in the treatment of heart disease [25–32].

### Exosome Cargo as Biomarkers for CVD

Biomarkers serve as critical tools for assessment of cardiovascular system health. Traditional biomarkers have included measuring cardiac troponins, such as cardiac troponin T (cTnT), which has served as the gold standard for MI diagnosis. However, it is not without its limitations, as cTnT levels can be elevated because of blood clots and cross-reactions with skeletal muscle troponins [33]. The other commonly used marker has been cytokines, but they lack specificity, making them difficult to use for CVD diagnosis [33]. Another traditional biomarker is C-reactive protein (CRP), a marker of CVD-induced inflammation, but it suffers from the same lack of specificity as cytokines. Therefore, more accurate biomarkers are needed to help with diagnosis. Emerging biomarkers include growth differentiation factor 15 (GDF15), cluster of differentiation 40 (CD40), interleukin 6 (IL- 6), and non-coding RNAs [34]. Non-coding RNAs are of particular interest as they are natural gene regulatory elements and are packaged in exosomes along with other cargo. Three commonly found exosome non-coding RNA cargo molecules include microRNAs, (miRNAs), long non-coding RNAs, (lncRNAs), and circular RNAs (circRNAs). These RNAs, along with other nucleic acid, protein and lipid cargo, all serve as potential biomarkers for CVD [35].

#### MicroRNAs

In recent studies, miRNAs have been highlighted as a key cargo component of exosomes with the ability to inform and protect against cardiac disease pathologies [36]. The potential of MSC-derived EVs and their protective capability was demonstrated in a daunorubicin induced cardiac microvascular endothelial cell injury model. This protection was facilitated by miR- 186 - 5p overexpression through its inhibition of the PARP9/STAT1 signaling pathway [37]. In another report, miRNA let- 7i- 5p was loaded into bone marrow-derived MSCs and tested for its therapeutic effects in MI. Ultimately, this miRNA played a protective role through inhibiting myocardial apoptosis, a critical factor in MI progression [38]. Additionally, miR- 744 - 5p was utilized in a similar manner as a novel treatment approach to mitigate myocardial inflammation and oxidative stress [39]. MSC exosome-derived miR- 21 - 5p was also shown to exert protective effects against MI by targeting the YAP1 signaling pathway [40]. These studies highlight the diverse roles of miRNAs and their potential power when harnessed with MSC-derived EVs. Notably, iPSCs also present a potential source of MSCs for CVD therapy. In a recent study, the cardioprotective effects of exosomes isolated from iPSC-MSCs were determined using a Doxorubicin (DOX)-induced cardiomyopathy mouse model. Exosomal microRNA sequencing revealed miR- 9- 5p was associated with protective effects, such as improvement of cardiac function and reduction of heart damage. These effects were accomplished through inhibition of the VPO1/ERK signaling pathway, which is implicated in cell damage and aging. This miRNA prevented mitochondria fragmentation and CM senescence due to DOX treatment [41].

MicroRNAs also mediate the effects of pharmacological CVD treatments. In fact, pretreating MSCs with vericiguat, a medication used for reduction of cardiovascular death risk, has been shown to enhance the therapeutic efficacy of MSC-derived exosomes through acting on the miR- 1180 - 3p/ETS1 pathway [42]. In another study, Danshen decoction (DSD), an herbal preparation used in Chinese medicine for the treatment of CVD, displayed protective effects against myocardial ischemia/reperfusion (I/R) injury by targeting miR- 93 - 5p and modulating the TXNIP/NLRP3/Caspase- 1 signaling pathway. This led to reduced myocardial pyroptosis and inflammation [43]. Exosomes derived from DSD pretreated bone marrow MSCs also showed therapeutic benefits for MI through apoptosis inhibition and promotion of CM autophagy, leading to improved cardiac function and fibrosis reduction [44]. These studies highlight how combinations of current medications and therapeutics on the market can be combined with new innovative treatment methods to enhance CVD management.

Cardiomyocytes and other cardiac tissues have been shown to release small EVs that carry specific miRNAs, which play crucial roles in cardiac remodeling while under stress. One such example is let- 7b- 5p. When interacting with TLR7, a receptor mediating immune and inflammatory responses, let- 7b- 5p activates the receptor, leading to adverse cardiac remodeling through the promotion of pro-inflammatory and pro-fibrotic responses [45]. In other cases, EVs carry bioactive molecules that induce protective effects. miR- 24 - 3p is another miRNA contained in EVs and secreted by CMs that reduces fibrosis in human cardiac microtissues through inhibition of pro-fibrotic pathways and collagen production by cardiac fibroblasts [46]. miR- 15b- 5p has been identified as a key player in reprogramming cardiac metabolism through impairment of mitochondrial function and energy production [47]. Other cell types such as macrophages release exosomes with miR- 155 that leads to inhibition of cardiomyocyte proliferation, thereby impairing heart regeneration [48]. Injured mouse cardiomyocytes also release exosomes that mediate cardiac proliferation and heart regeneration. miR- 21 - 5p is carried by such exosomes and acts on pathways such as ERK and AP- 1, encouraging cell cycle reentry [49]. Taken together, there is a large body of evidence from the last 1–2 years that suggests the undeniable potential of numerous miRNA cargo molecules in treating CVD (Table 1).

**Table 1:** Exosomal microRNA cargo origins, mechanisms, and clinical potential

miRNA(s)	Cell Origin	Exosome Derivation/Production	Clinical Potential	Signaling Mechanism of Action	Ref
miR- 126, miR- 21, miR- 133a, miR- 199	Lung progenitor cells	Engineered/Loaded	Treat acute MI	N/A	[24]
miR- 221, miR- 222	ADSCs	Derived	Mitigate PM-mediated I/R	BNIP3-MAP1LC3B-BBC3/PUMA	[26]
miR- 186 - 5p	Human MSCs	Derived	Mitigate anthracycline-induced endothelial injury	PARP9/STAT1	[37]
let- 7i- 5p	BMSCs	Derived	Protect against acute MI	Bax/Bcl- 2	[38]
miR- 744 - 5p	BMSCs	Engineered/Loaded	Suppress sleep apnea-induced CM damage	NFIX pathway	[39]
miR- 21 - 5p	MSCs	Derived	Protect against acute MI	Inhibit YAP1 pathway	[40]
miR- 1180 - 3p	MSCs	Derived	Reduce cardiac fibrosis	Inhibit ETS1 pathway	[42]
miR- 93 - 5p	BMSCs	Targeted	Treat I/R injury	TXNIP/NLRP3/Caspase- 1	[43]
let- 7b- 5p	NMCMs	Derived	Mediate cardiac remodeling	TLR7/MyD88/NF-κB	[45]
miR- 24 - 3p	iPSC-CMs	Derived	Reduce cardiac fibrosis	Inhibit FURIN/CCND1/SMAD4	[46]
miR- 15b- 5p	Primary cardiac tissue	Derived	Regulate CM metabolism	N/A	[47]
miR- 155	Macrophages	Derived	Inhibit CM proliferation	Inhibit IL- 6R/JAK/STAT3	[48]
miR- 21 - 5p	Primary cardiac tissue	Derived	Promote heart regeneration	Inhibit Spry- 1/PDCD4	[49]
miR- 302	BMSCs	Engineered/Loaded	Treat I/R injury	YAP pathway	[86]
miR- 25 - 3p	BMSCs	Engineered/Loaded	Treat I/R injury	Inhibit JAK2/STAT3	[89]
miR- 483 - 5p	ADSCs	Derived	Ameliorate DVT	Inhibit MAPK1/DRP1-NLRP3	[90]

Abbreviations: ADSC: Adipose-Derived Stem Cell; BMSC: Bone Marrow-Derived Mesenchymal Stem Cell; CM: Cardiomyocyte; DVT: Deep Vein Thrombosis; I/R: Ischemia/Reperfusion; iPSC: Induced Pluripotent Stem Cell; MI: Myocardial Infarction; miR: MicroRNA; MSC: Mesenchymal Stem Cell; NMCM: Neonatal Mouse Cardiomyocyte; PM: Particulate Matter

#### Long Non-Coding RNAs

Several recent studies have demonstrated the therapeutic potential of stem cell-derived exosomal lncRNAs in cardiac and vascular disease progression and regeneration. Intrapericardial injection of human umbilical cord MSC-derived EVs improved myocardial function in a mouse MI model. One of the EV cargo molecules, TARID (TCF21 antisense RNA inducing demethylation) was shown to upregulate *Tcf21* expression in epicardium-derived cells (EPDCs), which mitigated adverse remodeling after MI. Administration of TARID-laden nanoparticles to injured mouse and swine reduced infarct size, decreased collagen accumulation, and suppressed ventricular dilation, thereby revealing a novel target for improving cardiac fibrosis [50]. Other lncRNAs display similar results as administration of MSC-derived EVs to rats with heart failure leads to elevated levels of H19, a molecule associated with various cancers. After EV treatment, H19 binds to miR- 29b- 3p to elicit beneficial cardiac remodeling and improve heart function [51]. Another potential lncRNA therapy for cardiac fibrosis involves RMST (rhabdomyosarcoma 2-associated transcript), a relatively understudied transcript. This non-coding RNA is detrimental to heart function, as its expression is associated with cardiac fibrosis after MI. RMST silencing via short hairpin RNA (shRNA) reduced cardiac fibroblast proliferation and extracellular matrix (ECM) deposition in vitro and alleviated cardiac fibrosis in vivo. These effects are mediated by regulation of the lysyl oxidase (LOX) pathway, an extracellular copper-dependent enzyme that catalyzes the formation of cross-links within ECM proteins [52].

LncRNA molecules are also implicated in several other cardiovascular disease phenotypes. Expression of nudix hydrolase 6 (NUDT6), an antisense transcript targeting fibroblast growth factor 2 (FGF2), increases in tissue samples from patients with abdominal aortic aneurysm and carotid artery disease. Inhibition of *Nudt6* in mouse vascular disease models decreased the risk of atherosclerotic plaque rupture and improved the morphology of abdominal aortas. As expected, overexpression of *NUDT6* decreased FGF2 expression in vitro, leading to less proliferation and more apoptosis of human vascular smooth muscle cells. Therefore, lncRNAs affect both the heart and the vasculature in CVD [53]. Importantly, when loaded into exosomes, lncRNAs provide protection against doxorubicin-induced cardiotoxicity [54] and viral myocarditis [55]. In the former study, bone MSC-derived exosomes overexpressing lncRNA GHET1 suppressed pyroptosis after doxorubicin administration to cultured CMs and rats [54]. In the latter study, exosomes derived from M2 macrophages and containing lncRNA AK083844 promoted macrophage regulation towards the M2 phenotype. This polarization resulted in the attenuation of myocardial inflammation and cardiac dysfunction in mice with viral myocarditis [55].

#### Circular RNAs

Recent evidence suggests circRNA molecules have the potential to mitigate the progression of certain cardiac pathologies, such myocardial I/R injury and cardiomyocyte hypertrophy. CircRNA is a single-stranded, closed loop of non-coding RNA often found in circulating exosomes, and exhibiting a variety of roles as transcriptional regulators, microRNA sponges, and scaffolds for protein synthesis [56, 57]. It is well-known that exosomes from adipose-derived mesenchymal stem cells (ADSCs) can ameliorate cardiac damage post-MI [58]. CircRNA expression is important in this setting, as circ-Stt3b decreases cell death, reactive oxygen species (ROS) generation, and inflammation in hypoxic conditions. This molecule activates the circ-Stt3b/miR- 15a- 5p/GPX4 signaling axis to decrease ferroptosis, a form of iron-mediated cell death [59]. In another ferroptosis study, RNA sequencing and RNA pull-down were utilized to investigate differentially expressed circRNA molecules and their protein interactions after myocardial I/R. Notably, reduced levels of ferroptosis-associated circRNA (FECAR) were observed after MI. Overexpression of this molecule in mice suppressed I/R-induced MI and improved cardiac function by inhibiting ferroptosis. Mechanistically, FECAR binds to NAMPT (nicotinamide phosphoribosyltransferase), which increases NAMPT-dependent Sirtuin1 (Sirt1) expression. This, in turn, promotes the activity of FOXO1 (forkhead box protein O1), thereby increasing Fth1 (ferritin heavy chain 1) transcription and suppressing CM ferroptosis [60]. Interestingly, Circ DENND4C from urine stem cell-derived exosomes can also inhibit pyroptosis (inflammation-mediated cell death), thereby alleviating I/R injury in the rat kidney [61].

Circular RNAs mediate more than just specific cell death pathways to prevent myocardial damage. One report utilized adult mouse CMs to probe the effects of circRNA circUtrn on exercise-induced cardiac protection. RNA sequencing showed that circUtrn was upregulated in swimming-trained mouse CMs compared to non-exercising mice. This increase was associated with a beneficial outcome, as circUtrn expression was required for physiological cardiac hypertrophy in vivo, and its overexpression prevented myocardial I/R injury, pathological remolding, and subsequent cardiac distress [62]. In addition to I/R injury, circRNA molecules may be beneficial in heart muscle disorders, such as hypertrophic cardiomyopathy (HCM). Specific circRNAs have been isolated in certain disease states, including circZFPM2, which is highly upregulated in HCM patient tissues. To understand its effects in HCM regulation, circZFPM2 was knocked down in several in vitro CM models, promoting pathological hypertrophy, disruption of mitochondrial function, and apoptosis. On the other hand, CM-specific overexpression of circZFPM2 led to enhanced contractility in cardiac organoids derived from HCM patients [63]. While the overall body of knowledge is still in its infancy, the evolving interest in circRNAs presents another avenue of research into their potential restorative benefits for cardiac maladies such as I/R injury and HCM.

#### PIWI-Interacting RNAs

One final class of non-coding RNA that has gained notoriety over the past year is piRNA (PIWI-interacting RNA), a small single-stranded RNA that regulates gene expression and silences transposable elements. It is also an emerging biomarker for CVD, cancer, diabetes, and kidney disease [64]. piRNAs are garnering attention for their roles in aortic disease. One molecule, AVCAPIR, is significantly upregulated during aortic valve calcification, promoting the development of calcific aortic valve disease (CAVD). It appears this piRNA acts via an epigenetic mechanism, as it blocks the ability of its protein binding partners to demethylate their targets. This enhances the stability of these targets, thereby promoting valve calcification [65]. However, piRNAs can also benefit aortic disorders. In one recent study, PELNs (plant exosome-like nanovesicles) were isolated from green tea leaves, loaded with a complex containing the piRNA HAAPIR, and administered to mice with aortic dissection via oral gavage. Three weeks after nanovesicle introduction, treated mice displayed increased survival rates, decreased vascular width, and less elastic fiber rupture compared to controls. Mechanistically, HAAPIR ameliorated aortic dissection by decreasing MMP9 (matrix metalloproteinase 9) levels and increasing α-SMA (α-smooth muscle actin) levels, with Mef2D as a direct downstream target. This study introduces a novel piRNA for aortic disease and a unique delivery method via extracellular vesicles [66]. Notably, piRNAs have been implicated in more than just vascular disease, as various molecules are known to promote cardiac proliferation and regeneration [67], inhibit cardiac fibrosis [68], and induce CM necroptosis during I/R injury [69]. Further research is necessary to more thoroughly decipher the roles of piRNAs in cardiovascular disease.

### Current Challenges and Perspectives on Biomarkers for Pediatric Heart Disease

Due to their ease of isolation and delivery combined with the abundance of evidence suggesting a correlation with CVD, exosomes represent a promising biomarker that can be used to screen for heart disease. In the United States, a highly endorsed method of screening for CHD in newborns is pulse oximetry, where the oxygen saturation of the blood is monitored over time, with abnormal readings possibly representing a defect [70]. While it has been shown to detect ~ 50–75% of previously undiagnosed CHDs [71], this number varies depending on the severity of the deformity. In a recent study conducted in the United Kingdom, ~ 15% of critical CHD cases and ~ 67% of serious CHD cases were not detected by pulse oximetry [72]. In addition, these measurements cannot be acquired until after birth, and due to these drawbacks, a screening method that can reliably detect CHD in utero and postpartum is needed.

As mentioned above, stem cells provide a safe and abundant population of exosomes that are relatively easy to isolate and present a potential cell-free system for targeted compound delivery [21–23]. In the last ~ 5 years, several reports have demonstrated the therapeutic efficacy of > 100 different exosome cargo molecules. When trying to identify candidate biomarkers for pediatric CHD, their specificity, sensitivity, accessibility, and clinical relevance must be considered. On that note, microRNAs are quite stable and circulate at high levels in the blood [73]. Specific miRNAs are also associated with different types of CVD, including heart failure, acute MI, arrhythmias, and coronary artery disease [74, 75]. This feature would allow different miRNAs to distinguish between healthy and diseased individuals better than existing protein biomarkers, such as cTnT and CRP. As highlighted above, exosome-derived miRNAs have demonstrated beneficial cardioprotective effects in numerous pre-clinical CVD models. And perhaps most importantly, their presence in circulation in utero and involvement in various gestational disorders presents a valuable tool for the detection and intervention of CHD as early as possible [76].

One of the more significant implications of using miRNA derived from exosomes as biomarkers is the potential of designing and delivering patient-specific therapeutics. Specifically, for pediatric CVD, early detection allows for early intervention. Monitoring of exosomal cargo derived from maternal placental EVs can give insights into the potential diseases that occur during fetal development. This can help tailor personalized therapies as the placenta is the communication hub linking mother and fetus with EV effects on mother and fetal vital organs [77]. Maternal EVs have been shown to influence cardiac cell maturation and heart growth [78]. Exosomes are secreted by many cells including placental trophoblasts. In addition, placental and fetal exosomes carry signaling molecules that can drive heart development and CM maturation [77, 78]. Dysregulation in this process impairs normal cardiac function. In pregnancy, conditions like pre-eclampsia are reflected in exosome cargo, specifically miRNAs, and there are long-term complications associated with these conditions such as increased risk of congenital heart defects [79]. Exosomes and their cargo molecules provide a snapshot of these stressors offering early insights and warnings about future disease states. Utilizing exosomes as biomarkers presents several potential benefits, including a non-invasive early diagnostic tool, real-time monitoring of pregnancy outcomes enabling early intervention, mitigation of cardiovascular stress in utero, risk management for long-term outcomes, and a customized pediatric care plan based on exosome profiling. These applications will also open doors for exosomal stem cell-based therapies.

To ensure the most benefit for CVD therapy, we suggest stem cells as the ideal source of exosomal miRNA molecules, as they can self-renew, and their EVs are more likely to carry cargo that would promote cell and tissue growth and repair [21, 80]. In addition, due to their limitless differentiation potential, stem cell-derived exosomes would theoretically be useful for treating diseases throughout the body, including the cardiovascular system. Their ability to release paracrine signals to neighboring cells may even allow for cell-free delivery systems [81]. While stem-cell derived exosomes present new and exciting opportunities for CVD detection and treatment, their medical use imposes challenges that must be addressed. Due to size differences and heterogeneity, there is no universal protocol available for EV separation and purification, but techniques are in development to address this shortcoming. In addition, scale-up operations are not cost-effective using traditional isolation methods, a challenge that is compounded by a lack of specificity of the cell or tissue source from which the exosome originates [82, 83]. Moreover, while experimental evidence shows that exosome cargo can be manipulated with low immunogenicity, loading with high efficiency and minimizing alterations to targeting behavior remains a hurdle [84]. Finally, long-term exosome stability presents an additional challenge, as they are natural products that are subject to degradation during storage [85]. Therefore, further optimization of long-term storage is necessary to standardize their use in the clinic. Addressing these limitations will be essential to the potential use of exosomes as biomarkers and therapeutics for CVD.

On that note, stem-cell derived EVs can also be engineered to carry specific miRNA cargo and target specific cell types. In a recent study, MSC-derived exosomes loaded with miR- 302 and equipped with a CM-specific peptide were efficiently internalized by CMs, thereby reducing myocardial I/R injury and inflammation [86]. Stem cell-derived exosomes can be loaded with a range of bioactive cargo including miRNAs, non-coding RNAs, proteins, cytokines, anti-inflammatory molecules, lipids, metabolites, mRNAs, tRNAs, and DNA fragments [87, 88]. If combined with a unique method of delivery, such as inhalation, these exosomes can reach the blood stream and target the heart [24]. Therefore, a targeted delivery system can overcome some of the specificity and storage limitations described above and may be the most effective means to ensure exosome administration has the intended benefits for patients. Overall, we believe stem cell-derived exosomal miRNAs represent a novel, natural, and versatile avenue for CVD detection and therapy. Instead of being the sole means of early CHD detection, we suggest these biomarkers be utilized in conjunction with existing methods. Between exosome cargo derived from MSCs, pluripotent stem cells, and placental cells, a wide range of congenital defects can be diagnosed early in development, with confirmation by echocardiography after birth if necessary. MicroRNA molecules serve as effective biomarkers due to their abundance, stability, and specificity. And this system can be taken one step further through engineered exosomes with specific cargo miRNAs for a personalized medicine approach. Together, stem cell-derived exosomes and their associated miRNAs provide a naturally occurring platform that can be leveraged to benefit countless CHD and CVD patients.

## Key References

- Ellis BW, Ronan G, Ren X, Bahcecioglu G, Senapati S, Anderson D, Handberg E, March KL, Chang H-C, Zorlutuna P (2022) Human Heart Anoxia and Reperfusion Tissue (HEART) Model for the Rapid Study of Exosome Bound miRNA Expression As Biomarkers for Myocardial Infarction. Small. 10.1002/smll.202201330..
  – Findings from this study demonstrate the viability of microRNAs as biomarkers for myocardial infarction.
- Gu J, You J, Liang H, Zhan J, Gu X, Zhu Y (2024) Engineered bone marrow mesenchymal stem cell-derived exosomes loaded with miR302 through the cardiomyocyte specific peptide can reduce myocardial ischemia and reperfusion (I/R) injury. J Transl Med. https://doi.org/10.1186/s12967-024–04981-7.
  – Findings from this study suggest engineered exosomes carrying specific miRNA molecules can be used to treat CVD.
- Li J, Sun S, Zhu D, et al. (2024) Inhalable Stem Cell Exosomes Promote Heart Repair After Myocardial Infarction. Circulation. 10.1161/CIRCULATIONAHA.123.065005.
  – Findings from this study reveal inhalation as a novel method of stem cell-derived exosome administration.

## Data Availability

No datasets were generated or analysed during the current study.
